# Single-cell and spatial transcriptomics reveal transplant-associated T cells and myeloid cells in human liver transplantation

**DOI:** 10.3389/fimmu.2026.1745647

**Published:** 2026-02-04

**Authors:** Tianyang Zhou, Kun Guo, Chengyong Dong, Yuhui Wang, Jiajun Gao, Zhenyu Xu, Zhaoyun Shi, Zhiqi Jiao, Cong Xia, Ke Hu, Zhenming Gao

**Affiliations:** 1 Division of Hepatobiliary and Pancreatic Surgery, Department of General Surgery, The Second Hospital of Dalian Medical University, Dalian, Liaoning, China; 2 Organ Transplantation Center, The Second Hospital of Dalian Medical University, Dalian, Liaoning, China; 3 Department of Pathology, The Second Hospital of Dalian Medical University, Dalian, Liaoning, China; 4 Division of Gastrointestinal Surgery, Department of General Surgery, The Second Hospital of Dalian Medical University, Dalian, Liaoning, China; 5 Department of Urology, The Second Hospital of Dalian Medical University, Dalian, Liaoning, China

**Keywords:** graft rejection, liver transplantation, myeloid cells, single-cell RNA sequencing, spatial transcriptomics, T cells

## Abstract

**Background:**

Liver transplantation is the only effective way for end-stage liver disease. Rejection remains the leading cause of graft failure. The dynamic changes of intrahepatic immune cells involved in rejection are not completely understood.

**Methods:**

We integrated single-cell RNA sequencing and spatial transcriptomics (ST) to analyze graft tissues from multiple stages of human liver transplantation. ST enabled high-resolution mapping of immune cell states and spatial distribution within liver grafts.

**Results:**

We identified several transplantation-associated T cell (taT) subsets, including CD4^+^ effector-like T cells (Teff_like), CD8^+^ precursor exhausted T cells (Tpex), and CD8^+^ transitional effector-like T cells (tTeff_like). The CD4^+^ Teff_like subset highly expressed chemokines such as CCL3. The CD8^+^ tTeff_like subset represented an intermediate state transitioning from the CD8^+^ Tpex subset toward terminal exhaustion and was enriched in the immune activity pathway. Monocyte_PPARG, enriched in the rejection group, may be recruited by CD4^+^ Teff_like via the MIF-(CD74+CD44) pathway and subsequently promote CD8^+^ Tpex to tTeff_like differentiation through the ICAM1-LFA1 pathway. ST suggested that these immune subsets dominated the rejecting liver grafts.

**Conclusion:**

These findings highlight the potential roles and spatial distribution of taT subsets in rejecting liver grafts, providing insights into local immune regulation and the development of targeted therapeutic strategies.

## 1 Introduction

Liver transplantation remains the only effective treatment for end-stage liver disease (1, 2). However, immune rejection, particularly T cell-mediated rejection (TCMR), is the leading cause of graft failure (3, 4). Although immunosuppressive agents such as glucocorticoids, calcineurin inhibitors, and mammalian target of rapamycin (mTOR) inhibitors can mitigate rejection (5), 15–25% of recipients treated with tacrolimus still develop acute rejection (6). Current immunosuppressants primarily act by nonspecifically inhibiting T cell activation (7), which may disrupt immune homeostasis and cause side effects. In addition to T cells, antigen-presenting cells (APCs) have emerged as important regulators in graft rejection (8). Therefore, A comprehensive understanding of the intrahepatic immune landscape is essential for developing more effective and targeted immunotherapies.

Recent advances in single-cell RNA sequencing (scRNA-seq) and spatial transcriptomics (ST) have enabled high-resolution profiling of immune responses in various diseases (9, 10), whereas their application in liver transplantation remains limited. Peng et al. investigated peripheral blood longitudinally in post-transplantation patients, suggesting dynamic immune changes (11). Yang et al. used scRNA-seq and ST in rat liver transplantation models and identified intermediate monocytes as promoters of TCMR via the Resistin-CAP1 pathway (12). Cai et al. constructed an immune cell atlas of peripheral blood and liver tissue from recipients with or without rejection (13). Another study reported enrichment of CD8^+^ tissue-resident memory T cells (Trm) in rejecting liver grafts, suggesting their role in the rejection process (14).

However, most existing studies have examined peripheral blood or animal transplantation models, and direct characterization of the intrahepatic immune microenvironment in human graft tissue is limited. Many previous studies have also focused on a single post-transplant time point, which limits understanding of dynamic immune changes during the transplantation process. ST has advanced the study of tissue immune microenvironment, but it has been used infrequently in liver transplantation. In particular, high-resolution spatial platforms remain underused. To address these gaps, we integrated scRNA-seq data from human liver tissues collected at pre-, intra-, and post-transplant stages and applied high-resolution ST to rejected grafts. This approach provides a comprehensive temporal and spatial immune atlas and implies immune mechanisms associated with graft rejection and tolerance.

## 2 Method

### 2.1 Collection of scRNA-seq data and ST samples

ScRNA-seq data were collected from five datasets, including liver tissue samples from 23 individuals (**Supplementary Table S1**): 11 pre-transplant samples (donor livers) (15–17), 8 intraoperative samples (2 hours after portal perfusion) (16, 17), and 13 post-transplant samples (7 with rejection and 6 non-rejection) (13, 14). ST samples were obtained from liver tissues of three patients treated at the Division of Hepatobiliary and Pancreatic Surgery, Seconde Hospital of Dalian University between 2024 and 2025. All patients underwent acute rejection. In one case, the patient developed liver failure despite receiving intravenous pulse steroid therapy, and liver tissue was obtained following treatment. Samples were analyzed using the Visium HD platform (10x Genomics). Clinical details are summarized in **Supplementary Table S2**.

### 2.2 Quality control and integration of scRNA-seq data

Raw data were imported into Seurat (v5.1.0), and quality control was performed on each sample independently. Cells were excluded if they expressed <500 genes, >25% mitochondrial transcripts, or abnormally high gene counts based on quantile thresholds. Data was normalized using “NormalizeData”. Highly variable genes were identified by using “FindVariableFeatures” (nfeatures = 3500). Data were scaled and cell cycle effects regressed out using “ScaleData”. Principal component analysis (PCA) was performed using “RunPCA”. Batch-effect correction was performed using “RunHarmony” [dims = 1:20]. A shared nearest neighbor (SNN) graph was built using “FindNeighbors”, followed by clustering with “FindClusters”. Dimensionality reduction was performed using “RunUMAP” for Uniform Manifold Approximation and Projection (UMAP) (dims = 1:20, min.dist = 0.2, n.neighbors = 50). For subclustering analyses (CD4^+^, CD8^+^ T and myeloid cell subsets), preprocessing steps were repeated, including normalization, PCA (dims = 1:20), Harmony batch correction, and UMAP, followed by clustering with FindNeighbors and FindClusters.

### 2.3 Cell type annotation

Cell types were annotated based on canonical marker genes. For CD4^+^ and CD8^+^ T cell subsets, AddModuleScore was applied using gene sets from a previous study (18), and the canonical marker genes are listed in **Supplementary Table S3**.

### 2.4 Pseudotime trajectory, transcription factors, and cell-cell communication analysis

Cell differentiation trajectories were reconstructed using Monocle3 (19) (v1.3.7). Transcription factors (TFs) analysis was inferred by SCENIC (20) (v1.3.1). Cell-cell communication analysis was performed using CellChat (21) (v1.6.1).

### 2.5 Functional enrichment analysis

Pathway activity across cell subsets was assessed using gene set variation analysis (GSVA) (22) with 50 hallmark gene sets from MSigDB (v2024.1) (23). The average expression matrix was calculated using “AverageExpression”. Gene sets were loaded using GSEABase (v1.66.0), and pathway scores were computed with GSVA (v1.52.3). Gene Ontology (GO) enrichment analysis was performed using “clusterProfiler” (24) (v4.12.0) on differentially expressed genes (DEGs) identified from TFs target genes in the regulatory network.

### 2.6 Sample preparation

Formalin Fixed Paraffin Embedded (FFPE) tissue blocks were prepared following the Visium HD FFPE Tissue Preparation Guide (CG000684, 10x Genomics). RNA quality was evaluated based on DV200, defined as the percentage of RNA fragments longer than 200 nucleotides, with >30% considered high-quality.

### 2.7 Library preparation and sequencing

FFPE sections were mounted on blank slides for dewaxing, hematoxylin and eosin (H&E) staining, and imaging. Library preparation followed the Visium HD Spatial Gene Expression User Guide (CG000685, 10x Genomics), including probe hybridization, ligation, slide processing, probe release, extension, and library construction. Paired-end sequencing was performed on a NovaSeq X Plus system (Illumina) according to the manufacturer’s instructions. Raw FASTQ files and histological images were processed using Space Ranger (v3.0.0) with default settings. Space Ranger generated ST data at 2, 8, and 16 μm resolutions.

### 2.8 ST analysis

Raw Visium HD ST data were processed using Seurat. To balance transcriptomic information content and spatial resolution, data were analyzed at a bin size of 16 μm. Spatial bins with zero transcript counts were excluded prior to downstream analysis. Data were normalized and scaled using “SCTransform” (25). Subsequent analyses were performed using the same workflow as applied in the scRNA-seq analysis. Cell type annotation of spatial data was performed using Robust Cell Type Decomposition (RCTD) (26), with the scRNA-seq dataset generated in this study used as the reference. RCTD was run with default parameters, with doublet mode enabled (doublet_mode = “doublet”). The inferred cell type proportions for each spatial bin were used for downstream spatial analyses and visualization.

### 2.9 Multiplex immunofluorescence

Multiplex immunofluorescence (mpIF) was performed on paraffin-embedded liver tissue sections using a sequential staining system based on tyramide signal amplification (TSA). Tissues were fixed, dehydrated, embedded in paraffin, and sectioned at a thickness of 4 μm. Sections were deparaffinized, rehydrated, and subjected to antigen retrieval, followed by blocking of endogenous peroxidase activity and nonspecific binding. Primary antibodies were applied sequentially, with each cycle followed by incubation with HRP-conjugated secondary antibodies and development with TSA fluorophores. After each staining cycle, antibody stripping was performed to remove bound antibodies before the next round of staining. Nuclei were counterstained with DAPI, and sections were mounted using an antifade mounting medium. Whole slide imaging was performed using a digital slide scanner, and images from multiple staining rounds were registered and merged for subsequent analysis. The procedure was performed by Servicebio (Wuhan, China).

### 2.10 Statistical analysis

All statistical analyses were performed in R (v4.4.1). Data normality was assessed using the Shapiro-Wilk test. Group differences were evaluated using two-tailed unpaired Student’s t-tests. P-values were corrected for multiple comparisons using the Benjamini-Hochberg method. Statistical significance was defined as *P < 0.05, **P < 0.01, ***P < 0.001. Non-significant results are shown as ns.

## 3 Results

### 3.1 Single-cell landscape of human liver graft

**Figure 1:**
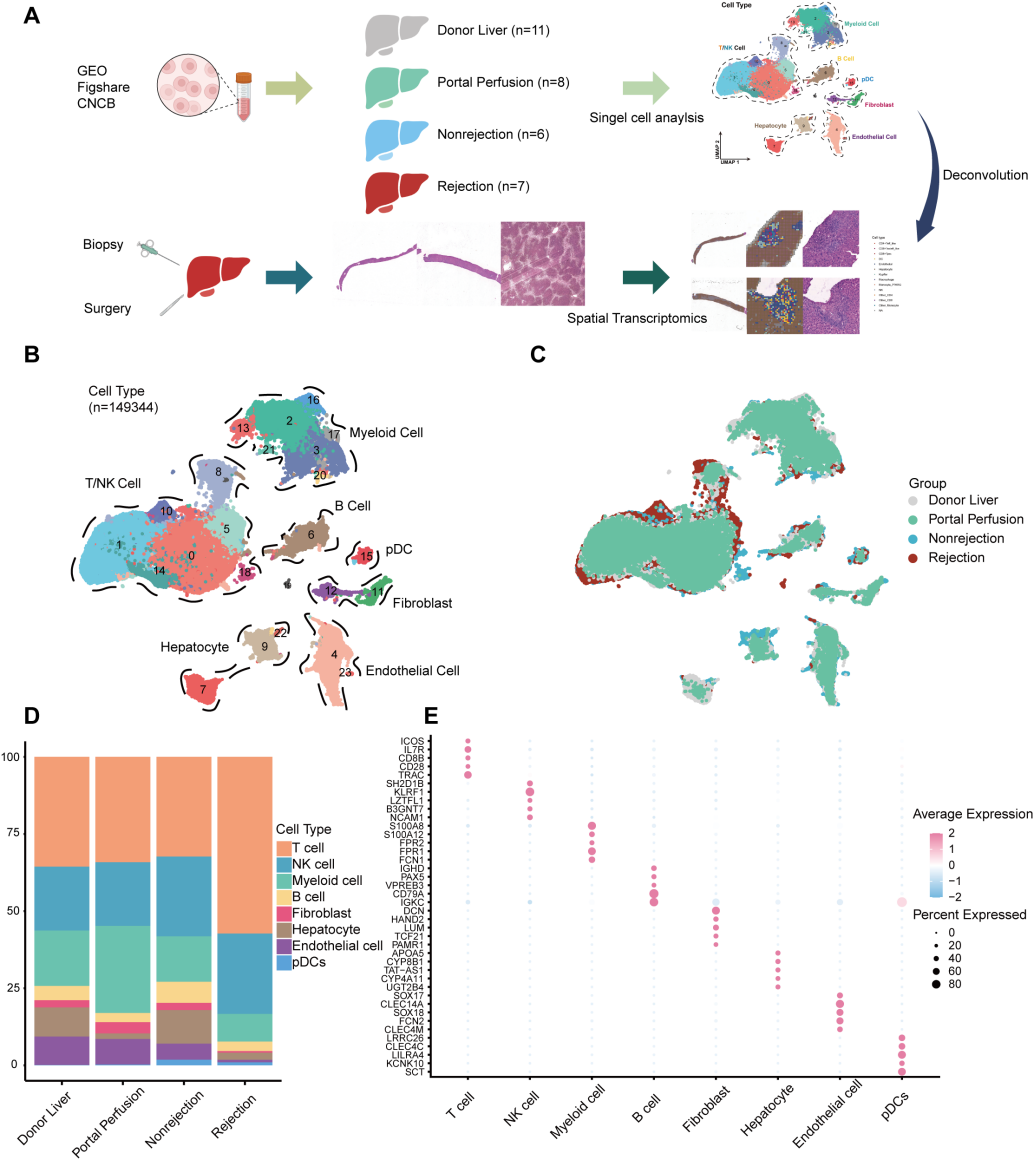
Construction of the single-cell landscape of human liver graft. **(A)** Schematic overview of the study workflow. **(B)** UMAP visualization of liver cell subsets colored by clusters. **(C)** UMAP visualization of single cells colored by group. **(D)** Bar plot showing the proportion of each cell type in different sample groups. **(E)** Dot plot displaying the top five DEGs for each cell type.

To characterize the cellular dynamics of liver graft tissue, we constructed a comprehensive single-cell atlas from 32 samples (**Figure 1**). A total of 149,344 high-quality single cells were analyzed and clustered into 23 subsets. Based on canonical marker genes, we identified eight major cell types: T cells, NK cells, B cells, hepatocytes, myeloid cells, endothelial cells, fibroblasts, and plasmacytoid dendritic cells (pDCs) (**Figure 1B**, **Supplementary Figure S1**). DEGs for each cell type are shown in **Figure 1E** and **Supplementary Table S4**. T cells, NK cells, and myeloid cells constituted the predominant immune compartments and exhibited distinct proportions across different groups (**Figures 1C, D**).

### 3.2 Identification of transplant-associated CD4^+^T cell subsets

**Figure 2:**
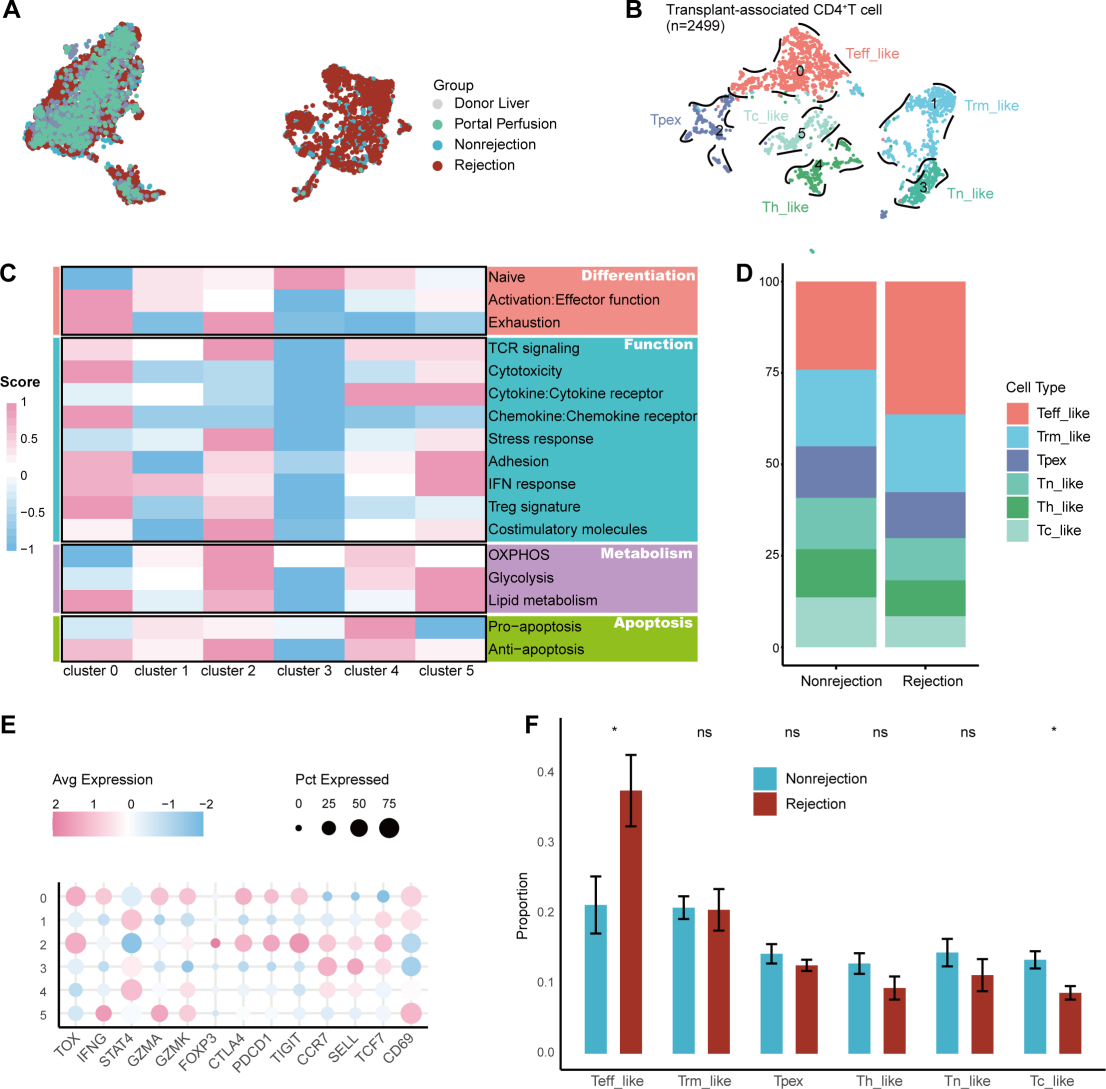
Single-cell transcriptomic analysis of taCD4^+^ T subsets. **(A)** UMAP visualization of CD4^+^ T subsets colored by group. **(B)** UMAP visualization of taCD4^+^ T subsets colored by clusters. **(C)** Heatmap of functional scores across subsets computed using AddModuleScore. **(D)** Bar plot showing the proportion of taCD4^+^ T subsets in rejection and non-rejection groups. **(E)** Dot plot showing marker gene expression for taCD4^+^ T subsets. **(F)** Proportional differences of taCD4^+^ T cell subsets between rejection and non-rejection groups.

We identified 13 distinct CD4^+^ T cell clusters (**Supplementary Figure S2A**). As shown in **Figure 2A**, several clusters were predominantly presented in either the rejection or non-rejection groups, suggesting they may represent transplant-associated CD4^+^ T cells (taCD4^+^ T). We then performed unsupervised clustering on these cells (**Figure 2B**). By using DEGs (**Supplementary Figure S2D**, **Supplementary Table S5**), module scores calculated with AddModuleScore (**Figure 2C**), and canonical marker genes (**Figure 2E**, **Supplementary Figure S2C**), we classified taCD4^+^ T cells into 6 representative transcriptional states.

Precursor exhausted T cells (Tpex, Cluster 2) expressed TOX, TCF7, IL7R, and PDCD1, consistent with their phenotype (27). (Teff_like, Cluster 0) exhibited high expression of CTLA4, PDCD1, TIGIT, and genes associated with cytotoxicity and inflammation, suggesting a dual phenotype of exhaustion and effector. Cytotoxic-like T cells (Tc_like, Cluster 5) were characterized by high expression of IFNG, GZMH, GZMK, and cytokine receptor genes. Helper-like T cells (Th_like, Cluster 4) expressed high levels of RORA and STAT. Tissue resident memory-like T cells (Trm_like, Cluster 1) exhibited features of both tissue resident and differentiated phenotypes. Naïve-like T cells (Tn_like, Cluster 3) expressed FHIT, CCR7, LEF1, SELL, and TCF7, consistent with a naïve CD4^+^ T cell phenotype (**Figures 2C, E**, **Supplementary Figure S2D**, **Supplementary Table S5**).

Comparative analysis suggested compositional differences in taCD4^+^ T cell subsets between the rejection and non-rejection groups (**Figure 2D**). The Teff_like subset was significantly enriched in the rejection group. Conversely, the other subsets were more abundant in the non-rejection group (**Figure 2F**).

### 3.3 Enhanced secretory function of CD4^+^ Teff_like cells

**Figure 3:**
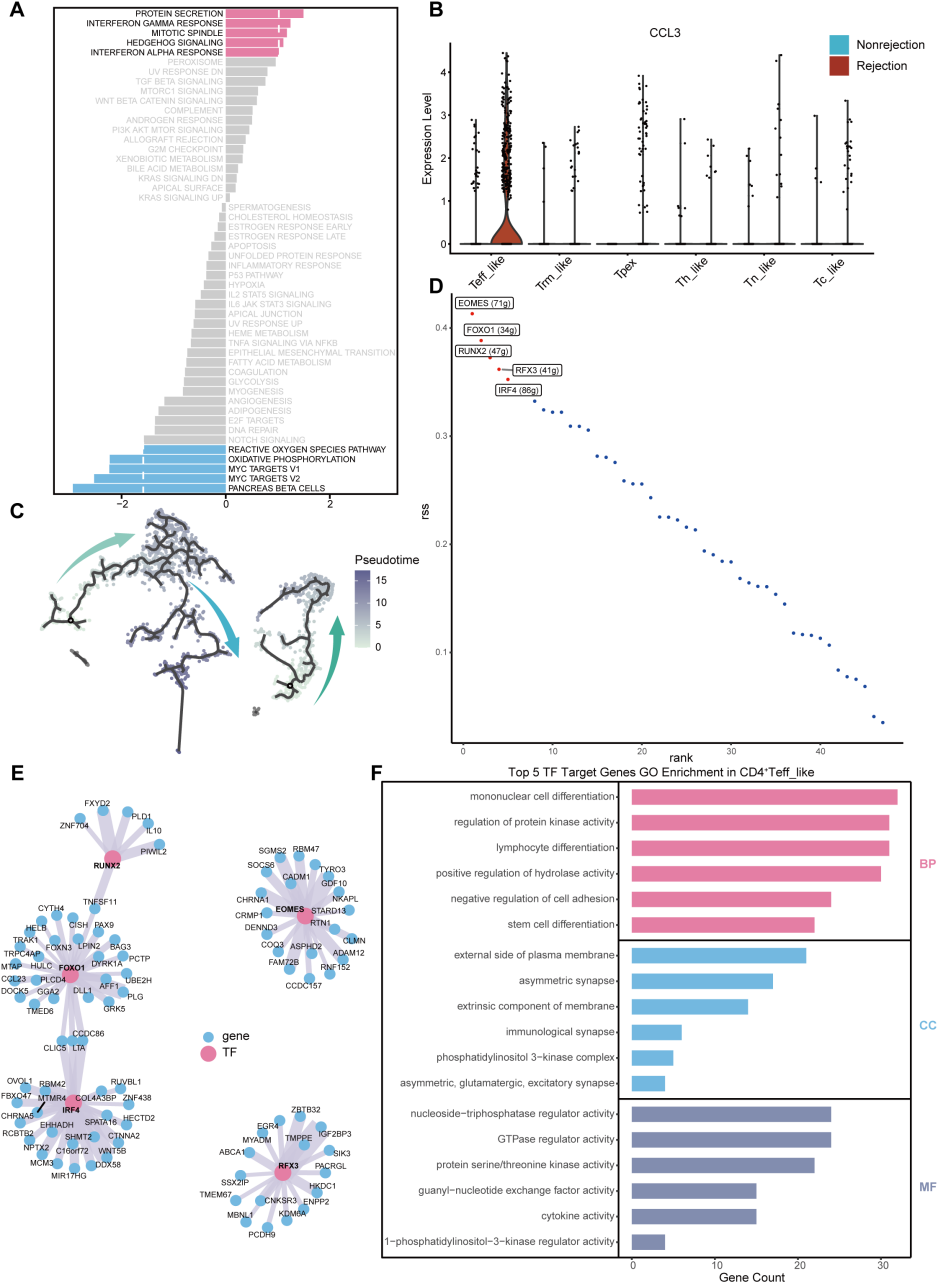
Functional analysis of the CD4^+^ Teff_like subset. **(A)** Differential pathway enrichment between CD4^+^ Teff_like and other taCD4^+^ T subsets based on GSVA scores. **(B)** CCL3 expression across taCD4^+^ T subsets, by group and cell type. **(C)** Pseudotime trajectory of taCD4^+^ T subsets. **(D)** Rank plot of RSS for TFs in the CD4^+^ Teff_like subset. **(E)** Regulatory networks of the top five TFs and their target genes in the CD4^+^ Teff_like subset. **(F)** GO enrichment analysis of target genes regulated by the top five TFs in the CD4^+^ Teff_like subset.

Given the enrichment of Teff_like subset in the rejection group, we performed an in-depth analysis of this subset. GSVA implied that the Teff_like subset was highly enriched in immune-related pathways, such as protein secretion compared to other taCD4^+^ T subsets (**Figure 3A**). The Teff_like subset exhibited high expression of chemokines CCL3, CCL4, and CCL5 (**Figure 3B**, **Supplementary Figures S3B, C**, **Supplementary Table S5**), suggesting that this subset may recruit other immune cells by chemokine secretion and contribute to graft rejection.

Pseudotime analysis showed that the Teff_like subset originated from the Tpex subset and appeared in the early to intermediate phases (**Figure 3C**). Consistently, CCL3, CCL4, and CCL5 reached peak expression at corresponding timepoints (**Supplementary Figure S4A**).

To identify key TFs driving the Teff_like phenotype, we ranked TFs activity based on regulon specificity scores (RSS). The top five TFs were EOMES, FOXO1, RUNX2, RFX3, and IRF4 (**Figure 3D**), suggesting their potential roles in regulating the functional state of this subset. We constructed the regulatory networks for these TFs (**Figure 3E**), suggesting their downstream target genes. Then, we performed GO enrichment analysis on their target genes. The results showed significant enrichment in immune-related processes including monocyte differentiation, lymphocyte differentiation, and regulation of protein kinase activity (**Figure 3F**, **Supplementary Table S6**).

### 3.4 Enrichment of CD8^+^ T cells exhibiting exhaustion features in the rejection group

**Figure 4:**
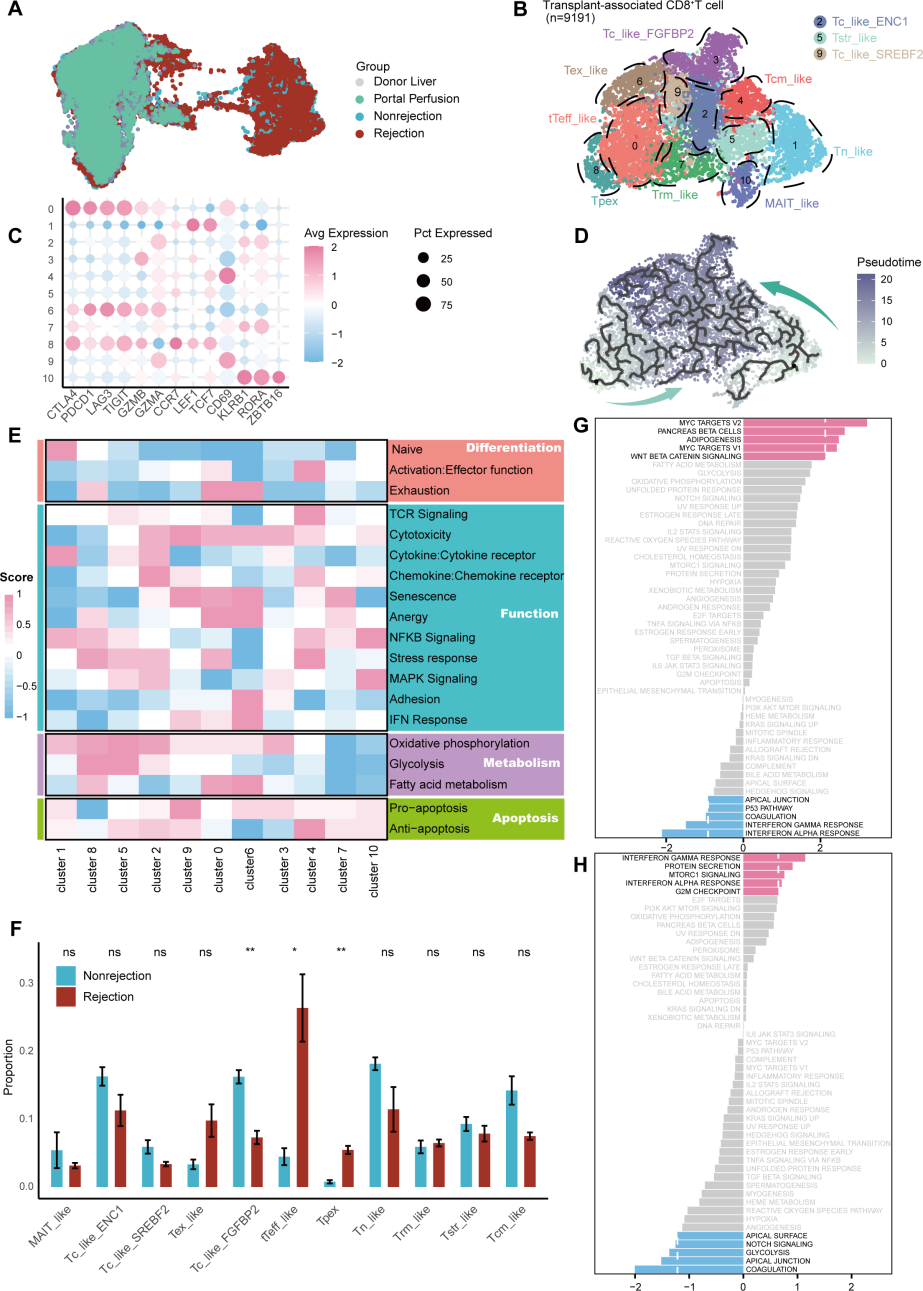
Single-cell transcriptomic analysis of taCD8^+^ T subsets. **(A)** UMAP visualization of CD8^+^ T cell subsets colored by group. **(B)** UMAP visualization of taCD8^+^ T subsets colored by clusters. **(C)** Dot plot showing marker gene expression across taCD8^+^ T subsets. **(D)** Pseudotime trajectory of taCD8^+^ T subsets. **(E)** Heatmap of functional scores across subsets computed using AddModuleScore. **(F)** Proportional differences of taCD8^+^ T subsets between rejection and non-rejection groups. **(G)** Differential pathway enrichment between the CD8^+^ Tpex subset and other taCD8^+^ T subsets based on GSVA scores. **(H)** Differential pathway enrichment between the CD8^+^ tTeff_like subset and other taCD8^+^ T subsets based on GSVA scores.

We analyzed CD8^+^ T cells (**Supplementary Figure S5A**) and identified transplant-associated T cell (taT) subsets predominantly presented in either the rejection or non-rejection group, similar to CD4^+^ T cells (**Figure 4A**). Unsupervised clustering implied 9 functional states among transplant-associated CD8^+^ T cells (taCD8^+^ T) (**Figure 4B**, **Supplementary Table S7**), including Tpex (Cluster 8), transitional effector-like T cells (tTeff_like, Cluster 0), Tc_like (Clusters 2, 3, 9), central memory-like T cells (Tcm_like, Cluster 4), tissue resident memory-like T cells (Trm_like, Cluster 7), stress-like T cells (Tstr_like, Cluster 5), exhausted-like T cells (Tex_like, Cluster 6), Tn_like (Cluster 1), and mucosal associated invariant-like T cells (MAIT_like, Cluster 10).

**Figure 5:**
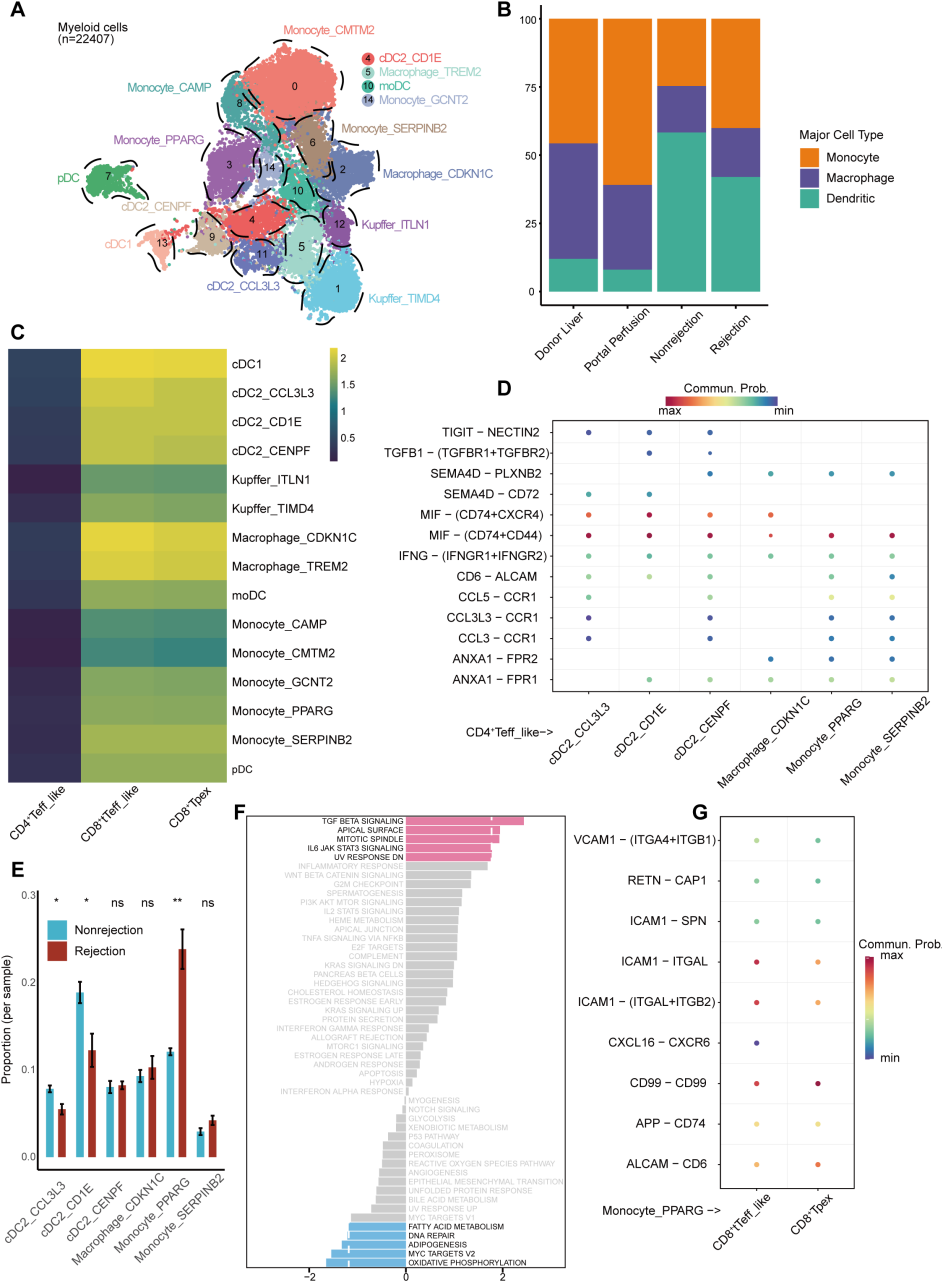
Myeloid subsets analysis and cell-cell communication. **(A)** UMAP visualization of myeloid subsets colored by clusters. **(B)** Bar plot showing the relative abundance of major myeloid subsets across different groups. **(C)** Communication strength between myeloid cells (senders) and selected taT subsets (receivers). **(D)** Non-MHC ligand-receptor interactions between the CD4^+^ Teff_like subset (senders) and selected myeloid subsets (receivers). **(E)** Proportional differences of selected myeloid subsets in rejection and non-rejection groups. **(F)** GSVA pathway enrichment scores comparing Monocyte_PPARG with other myeloid subsets. **(G)** Non-MHC ligand-receptor interactions between Monocyte_PPARG (senders) with CD8^+^ Tpex and CD8^+^ tTeff_like subsets (receivers).

The Tpex subset showed a precursor exhaustion phenotype with high TOX, TCF7, IL7R, and PDCD1 expression (**Figure 4C**, **FigureS5C**). The tTeff_like subset co-expressed PRF1, GZMB, exhaustion markers (CTLA4, PDCD1), and early activation markers (CD44, CD69), suggesting a transitional state with mixed function (28). The Tc_like subsets expressed high expression of cytotoxic genes (**Figures 4C**). The Tcm_like subset expressed GZMA, GZMK, CD44, and CD69, while downregulating activation markers such as NKG7 and PRDM1, consistent with a central memory phenotype. The Trm_like subset showed elevated expression of CD44, CD69, and ITGA1, along with reduced CCR7 and SELL, aligning with tissue-resident characteristics. The Tstr_like subset lacked canonical marker genes but exhibited high stress-related signaling and increased NF-κB pathway activity (**Figures 4C, E**), a key regulator of cellular stress responses (29). The Tex_like subset resembled the tTeff_like subset but had lower expression of early activation genes (CD44, CD69) and appeared at later pseudotime stages (**Figure 4D**). The Tn_like subset expressed naïve-associated genes at high levels (**Figures 4C, E**). The MAIT_like subset expressed canonical MAIT markers, including KLRB1, RORA, and ZBTB16.

To identify the key taCD8^+^ T subsets in the rejection group, we compared their proportions between rejection and non-rejection groups. Tpex and tTeff_like subsets were significantly enriched in the rejection group (**Figure 4F**, **Supplementary Figure S5D**). GSVA showed that the Tpex subset was associated with MYC and WNT/β-catenin pathways, suggesting strong proliferation and stem-like traits (**Figure 4G**). The tTeff_like subset exhibited activation of IFN responses, protein secretion, mTORC1 signaling, and G2M checkpoint pathways (**Figure 4H**).

Pseudotime analysis indicated a differentiation trajectory from Tpex to tTeff_like and finally to Tex_like subsets (**Figure 4D**). This dynamic process suggests that the Tpex subset may differentiate into the tTeff_like subset under specific stimulation to exert effector functions and promote rejection, instead of directly entering terminal exhaustion. Additionally, TIGIT and CTLA4 showed bimodal expression, peaking at early and late pseudotime stages (**Supplementary Figures S5E, F**).

### 3.5 Monocyte_PPARG subset activates CD8^+^ Tpex subset via ICAM1 - (ITGAL+ITGB2) interaction

We next focused on myeloid cells (**Figure 5A**), with their marker gene expression shown in **Supplementary Figure S6C** and **Supplementary Table S8**. No transplant-associated myeloid subsets were identified, unlike in T cells (**Supplementary Figure S6A**). However, compositional differences were observed between groups. Monocytes and macrophages were enriched in donor liver and portal perfusion groups, while dendritic cells (DCs) predominated in both rejection and non-rejection groups (**Figure 5B**).

Considering the effector functions and significant enrichment in the rejection group, we focused on the CD4^+^ Teff_like, CD8^+^ Tpex, and CD8^+^ tTeff_like subsets for intercellular communication analysis. Due to the APCs role of myeloid subsets (30), we first defined them as senders and evaluated their signaling towards CD4^+^ Teff_like, CD8^+^ Tpex, and CD8^+^ tTeff_like subsets (**Figure 5C**). Myeloid subsets showed weak interaction with the CD4^+^ Teff_like subset but stronger signaling to CD8^+^ Tpex and tTeff_like subsets. Given the ability of CD4^+^ T cells to recruit other immune cells by secreting chemokines (31) and the high cytokine-secreting function exhibited by the CD4^+^ Teff_like subset, this subset is more likely to act as signal senders rather than receivers in this network. Then, we assessed signaling from CD4^+^ Teff_like cells to myeloid subsets (**Supplementary Figure S6E**). 6 myeloid subsets with high interaction scores were selected for ligand-receptor analysis: conventional dendritic cell type 2 (cDC2)_CCL3L3, cDC2_CD1E, cDC2_CENPF, Macrophage_CDKN1C, Monocyte_PPARG, and Monocyte_SERPINB2. The dominant ligand-receptor pair was MIF-(CD74+CD44) (**Figure 5D**).

Then, we compared the abundance of these myeloid subsets between rejection and non-rejection groups. Monocyte_PPARG was significantly enriched in the rejection group (**Figure 5E**). GSVA suggested TGF-β and IL6-JAK-STAT3 pathway enrichment, indicating both pro-inflammatory and regulatory properties (**Figure 5F**). We further analyzed Monocyte_PPARG interactions with CD8^+^ Tpex and tTeff_like subsets. The main interaction was ICAM1-(ITGAL+ITGB2) (**Figure 5G**).

Since CD4^+^ T cells are involved in the activation of CD8^+^ T cells (32), we also examined signaling from CD4^+^ Teff_like to CD8^+^ Tpex and tTeff_like subsets (**Supplementary Figure S6D**). Although VCAM1-(ITGA4+ITGB1) and LCK-(CD8A+CD8B1) showed high interaction probabilities, they are involved in endothelial-immune cell adhesion (33) or intracellular T cell signaling (34).

### 3.6 ST implies dominance of taT subsets in rejection

To address the limited resolution of conventional Visium (~55 μm bins containing 1–10 cells), we applied the high-resolution Visium HD platform (10x Genomics), released in 2024. ST was performed on liver biopsy from two patients with acute rejection, and on a postoperative pathological specimen from a third patient who required re-transplantation due to liver failure despite intravenous pulse steroid therapy.

**Figure 6:**
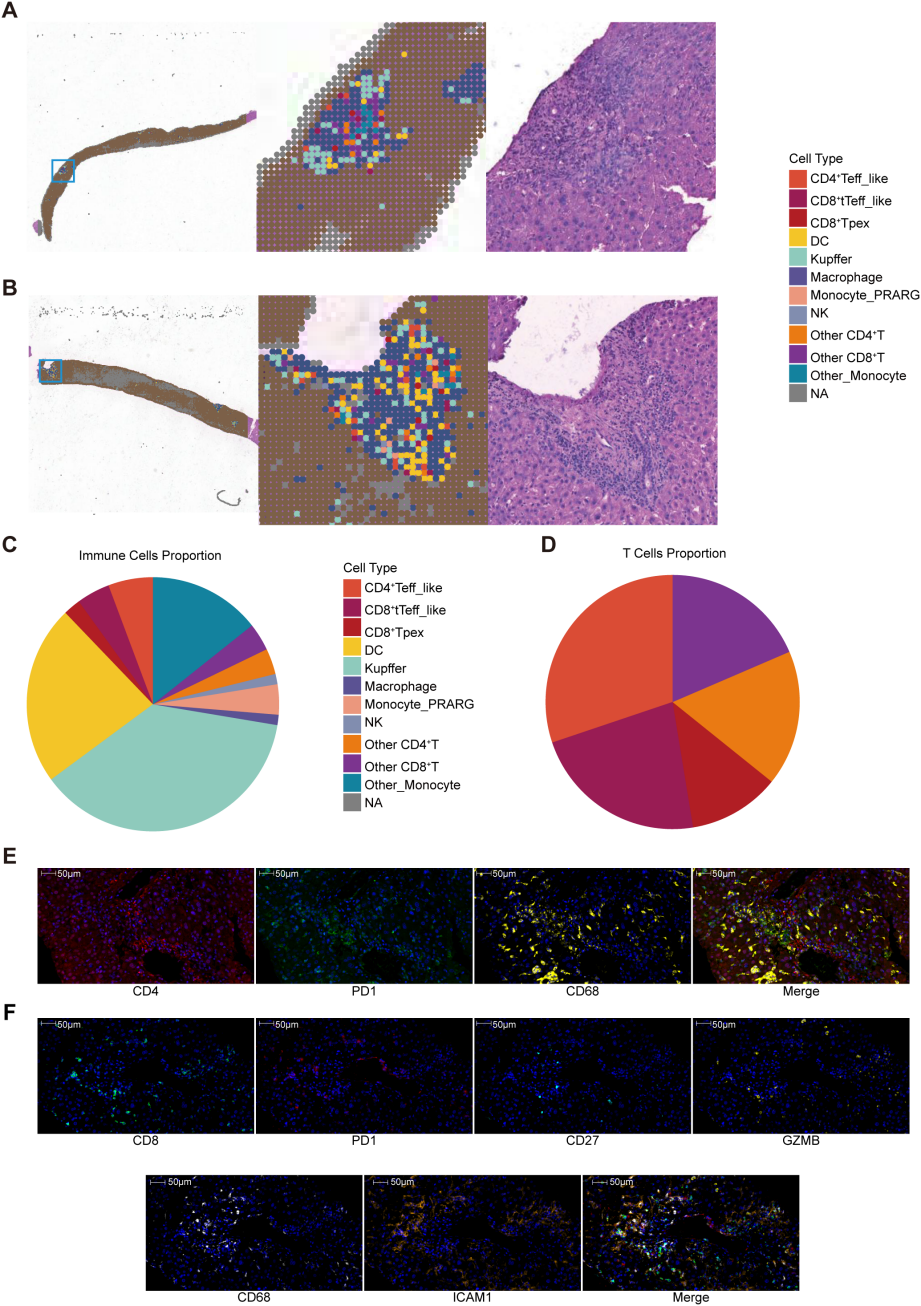
ST analysis of liver graft rejection. **(A)** Deconvolution results for Patient 1. **(B)** Deconvolution results for Patient 2. **(C)** Pie chart showing the proportions of immune cell types. **(D)** Pie chart showing the proportions of T cell subsets. **(E)** MpIF showing the spatial distribution of CD4, PD1, and CD68. **(F)** MpIF showing the spatial distribution of CD8, PD1, CD27, GZMB, CD68 and ICAM1.

As canonical marker genes were insufficient for accurate cell type annotation in Visium HD data (**Supplementary Figure S7E**), we used our study’s scRNA-seq data as a reference for deconvolution-based cell type annotation. This approach enabled high-resolution mapping of immune and structural cells in liver tissue. In representative samples (Patient 1 and Patient 2), immune cells were predominantly localized around the portal area (**Figures 6A, B**, **Supplementary Figures S8A–C**). Across all samples, Kupffer cells and DCs were the dominant myeloid subsets (**Figure 6C**), while CD4^+^ Teff_like and CD8^+^ tTeff_like subsets dominated T cell subsets (**Figure 6D**, **Supplementary Figures S8D–F**).

In Patient 3, tissue damage resulted in fewer annotated bins. Residual immune cells were mainly located near the portal area margins and hepatic sinusoids (**Supplementary Figures S8A–C**). Neighborhood analysis suggested a higher spatial proximity between Monocyte_PPARG and CD8^+^ Tpex subsets compared to other T cell subsets, supporting the feasibility of local myeloid-T cell interactions within rejection-associated niches. (**Supplementary Figure S8G**).

To validate the ST findings at the tissue level, mpIF was performed on human rejection grafts. CD4 and PD1 were mainly distributed in the portal area, while CD68 was located around the portal area and within hepatic sinusoids (**Figure 6E**). CD68 and ICAM1 showed a colocalized distribution (**Figure 6F**, **Supplementary Figure S8H**). CD8 was observed as focal aggregates in the portal area and was accompanied by CD27 and GZMB expression, with partial overlap with PD1 signals (**Figure 6F**, **Supplementary Figure S8H**).

## 4 Discussion

In this study, we integrated scRNA-seq and ST analyses to comprehensively characterize immune cell states in liver tissues across different stages of transplantation. We identified several taT subsets that emerged post-transplantation, among which CD4^+^ Teff_like and CD8^+^ tTeff_like subsets were significantly enriched in the rejection group, suggesting their potential roles in mediating graft rejection. Unlike previous studies that focused solely on post-transplantation, our data integrated pre-, intra-, and post-transplantation phases, providing a dynamic and clinically relevant view of the evolving intrahepatic immune landscape.

The CD4^+^ Teff_like subset exhibited high levels of chemokine genes, such as CCL3 and CCL4, suggesting their potential role in mediating immune cell recruitment during graft rejection (35). SCENIC analysis implied increased activity of key TFs, including IRF4, RUNX2, and EOMES. Notably, IRF4 is a critical regulator of T cell activation (36), and a previous study has implicated it in promoting graft rejection (37). In our analysis, IRF4 target genes were enriched in monocyte differentiation pathways, suggesting that IRF4 may mediate interactions between the CD4^+^ Teff_like subset and monocytes.

CD8^+^ T cell analysis suggested a differentiation trajectory from Tpex to tTeff_like, and eventually to Tex_like cells. This dynamic progression suggested that the CD8^+^ Tpex subset may first undergo activation and clonal expansion before gradually entering a state of exhaustion. We observed that the tTeff_like subset was relatively enriched in the rejection group and showed upregulation of inflammatory and activation pathways, including interferon responses, mTOR signaling, and protein secretion. These findings suggest that the tTeff_like subset represented a functionally active intermediate. Previous studies have reported an increased presence of exhausted T cells in liver grafts undergoing rejection (38), and that CD8^+^ Tpex cells can give rise to transitional effector cells (39). Our study found that the CD8^+^ tTeff_like subset exhibited both exhaustion-related markers and strong effector function.

The absence of transitional T cells between non-transplant and post-transplant groups in our analysis likely reflects the clinical sampling context rather than a true lack of such states *in vivo*. Pre- and intra-transplant liver tissues predominantly contain donor-derived resident immune cells, whereas post-transplant biopsies are largely composed of recipient-derived, alloantigen experienced infiltrating T cells. The transitional phase between these immune environments may be transient and therefore difficult to capture without high-frequency longitudinal liver sampling, which is rarely feasible in clinical practice. Future time-resolved sampling or experimental transplantation models will be important for resolving these dynamic intermediate populations.

The pronounced changes in CD4^+^ and CD8^+^ T cell subsets after transplantation likely reflects the central role of T cell activation and clonal expansion in T cell-mediated rejection (40). Sustained alloantigen stimulation may drive strong adaptive immune responses, and standard post-transplant immunosuppressive therapy predominantly targets T-cell activation and proliferation (41), leading to marked transcriptional and functional diversification of T-cell populations. In contrast, myeloid cells are less directly affected by immunosuppression and may respond mainly through changes in activation state rather than major restructuring of subset identity (42). This biological distinction may explain why T-cell subsets displayed strong separation across conditions, whereas myeloid clustering patterns remained relatively stable.

Although no transplant-associated myeloid subset was identified, differences in the proportions of myeloid subsets were observed across groups. In donor liver and portal perfusion groups, monocytes and macrophages predominated, likely reflecting innate immunity triggered by brain death and surgical stress (43). In contrast, DCs became more abundant post-transplantation, consistent with the activation of adaptive immunity (44).

The Monocyte_PPARG subset was enriched in the rejection group and upregulated TGF-β and IL6-JAK-STAT3 signaling, suggesting dual pro-inflammatory and regulatory potential (45, 46). Cell-cell communication analysis implied that Monocyte_PPARG subset received signals from CD4^+^ Teff_like cells via the MIF-(CD74+CD44) pathway, then interacted with CD8^+^ Tpex and tTeff_like subsets through the ICAM1-(ITGAL+ITGB2) pathway. Macrophage migration inhibitory factor (MIF) promotes immune cell recruitment and retention within inflamed tissues, primarily through its receptor CD74 (47). ITGAL and ITGB2 encode CD11a and CD18, respectively, which form lymphocyte function-associated antigen-1 (LFA-1) (48). ICAM1 binds LFA-1 to promote immunological synapse formation and enhance T cell activation (49). These findings suggested a cross-lineage interaction model involving CD4^+^ T cells, monocytes, and CD8^+^ T cells. Molecules along this axis may serve as therapeutic targets to mitigate rejection and promote immune tolerance.

ST analysis suggested immune cell enrichment around portal areas, consistent with hepatic anatomical structure (50). Notably, taT subsets were concentrated in rejecting samples. A similar pattern was observed after steroid therapy, with residual immune cells clustering around portal areas and hepatic sinusoids, suggesting these regions serve as niches sustaining rejection. Patient 3’s liver sample exhibited a relatively large portal area and a higher proportion of CD4^+^ Teff_like cells than the other two biopsy samples. Given the known functional compartmentalization of hepatic immunity (51), this finding indicated potential spatial heterogeneity in the liver during rejection. Moreover, we observed a closer spatial proximity between the Monocyte_PPARG and CD8^+^ Tpex subsets. MxIF analysis showed a colocalization of CD8^+^ T cells with CD68^+^ ICAM1^+^ myeloid cells in the portal region.

High-resolution ST is critical in the context of liver transplant rejection. In clinical practice, liver biopsies rather than surgical specimens represent the primary and most realistic source of rejecting graft tissue, as re-transplantation is rare and most patients receive steroid pulse therapy before any surgical intervention. Consequently, biopsy samples are small and often contain limited portal areas, which are essential for the histological assessment of T cell-mediated rejection. Conventional spatial transcriptomic platforms with a 55-µm spot size risk capturing entire portal areas within only a few spots, making it difficult to resolve local immune heterogeneity or accurately evaluate cellular interactions in the rejection microenvironment. The high-resolution platform enables the precise mapping of immune cell localization within portal areas, facilitating detailed analysis of the underlying mechanisms of rejection. This approach is therefore not only technically advantageous but also clinically necessary to faithfully characterize the intrahepatic immune landscape in human liver transplant rejection.

Our study has several limitations. First, the scRNA-seq sample size was limited, potential confounders such as primary diseases were not stratified. Additionally, the ST data were only available for the rejection group, without non-rejection controls included. Second, our findings were mainly based on transcriptomic data and lacked validation at the functional level. Future studies integrating spatial proteomics, flow cytometry, or other functional assays will be essential to further confirm the identity of key cell subsets and their interaction patterns.

Previous studies have shown that the TCR repertoire in peripheral blood differs markedly from that within liver tissue post-transplantation (52), indicating the importance of characterizing the intrahepatic immune environment in liver transplantation. Existing animal models often use strain-mismatched transplants without immunosuppression, limiting their clinical relevance (53). In contrast, our study exclusively utilized human liver tissue samples, covering multiple stages, including pre-, intra-, and post-transplantation phases. This provided a more clinically relevant view compared to previous studies that primarily focused on peripheral blood or animal models.

## Data Availability

The raw sequence data of Visium HD reported in this paper have been deposited in the Genome Sequence Archive (Genomics, Proteomics & Bioinformatics 2025) in National Genomics Data Center (Nucleic Acids Res 2025), China National Center for Bioinformation / Beijing Institute of Genomics, Chinese Academy of Sciences (GSA-Human: HRA022383).
