# Supplementary material for: Single-cell and spatial transcriptomics reveal transplant-associated T cells and myeloid cells in human liver transplantation
Source: Front Immunol. 2026 Feb 4;17:1745647. doi: 10.3389/fimmu.2026.1745647 (PMC12913157; doi:10.3389/fimmu.2026.1745647)
Supplement: Supplementary file 1 [file Image1.pdf]

Supplementary Fig. 1

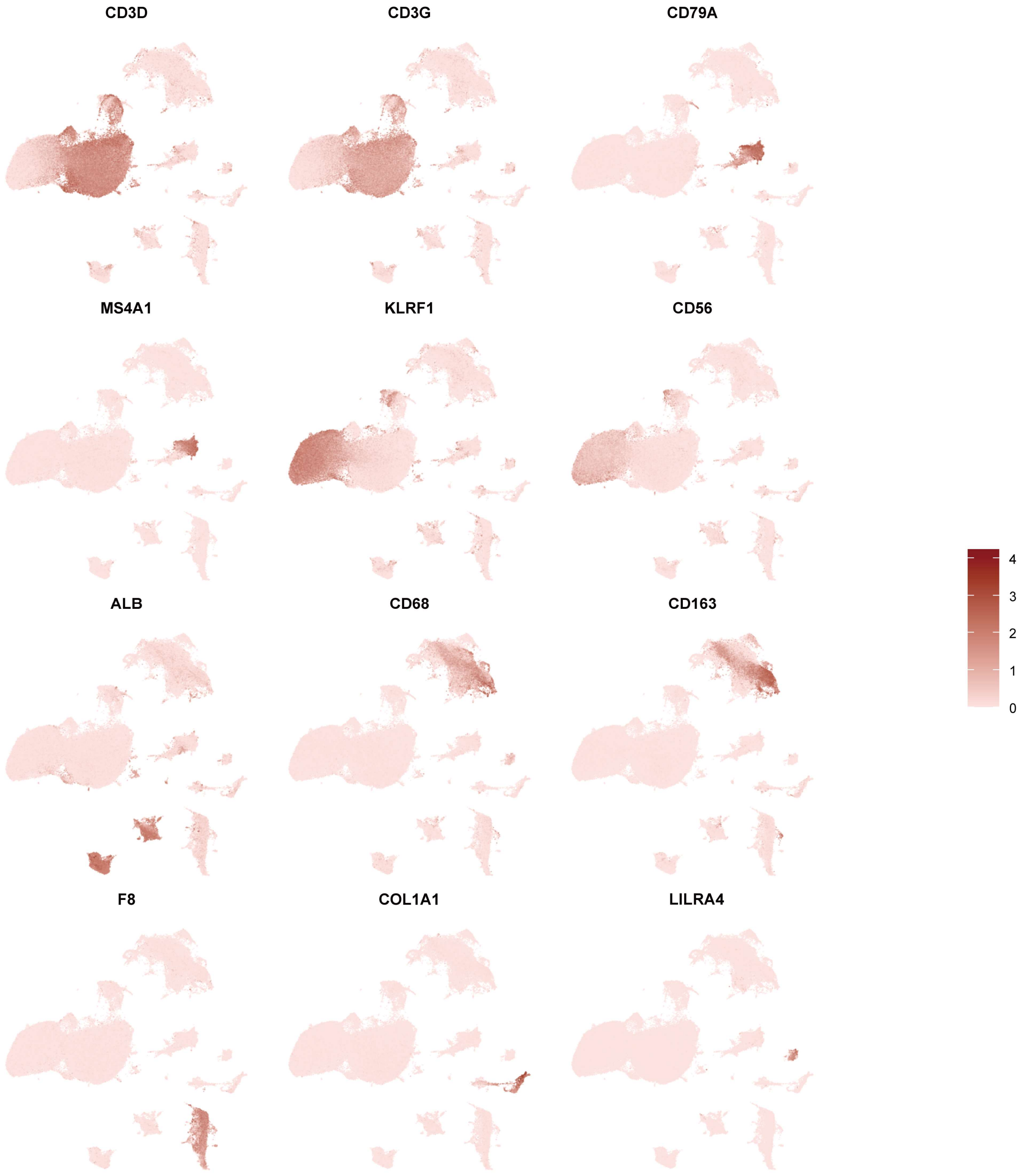

Supplementary Figure 1.

UMAP visualization of the expression of canonical marker genes used to identify major cell types: T cells (CD3D, CD3G), B cells (CD79A, MS4A1), NK cells (KLRF1, CD56), hepatocytes (ALB), myeloid cells (CD68, CD163), endothelial cells (F8), fibroblasts (COL1A1), and pDCs (LILRA4).

Supplementary Fig. 2

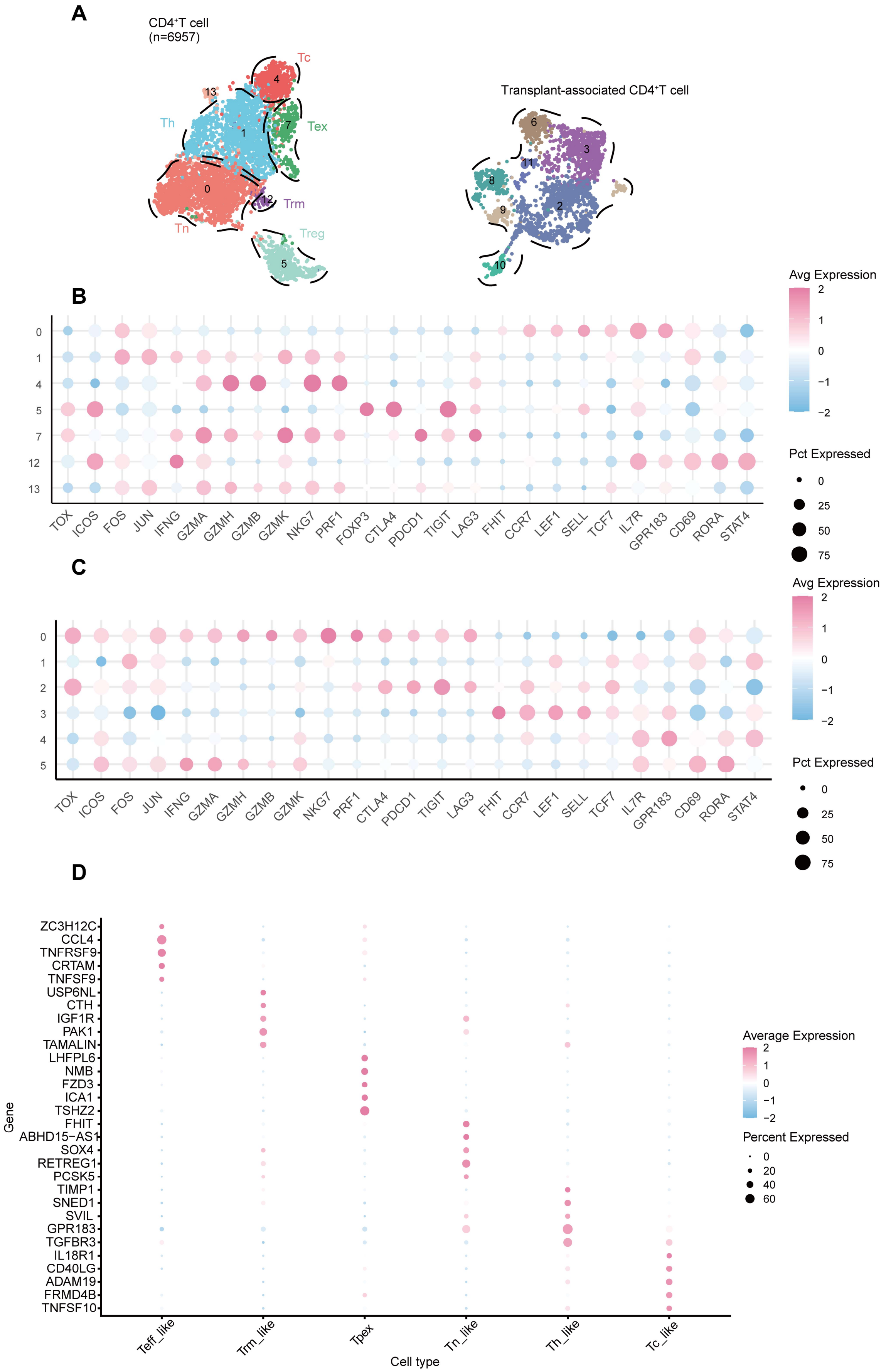

Supplementary Figure 2.

(A) UMAP visualization of $CD4^+$ T cells colored by clusters. Cluster 13 expressed hepatocyte-specific marker genes and was therefore considered to represent contamination.

(B) Dot plot showing the expression of canonical $CD4^+$ T cell marker genes.

(C) Dot plot showing the expression of more canonical $taCD4^+$ T subsets marker genes.

(D) Dot plot displaying the top 5 DEGs in $taCD4^+$ T subsets.

Supplementary Fig. 3

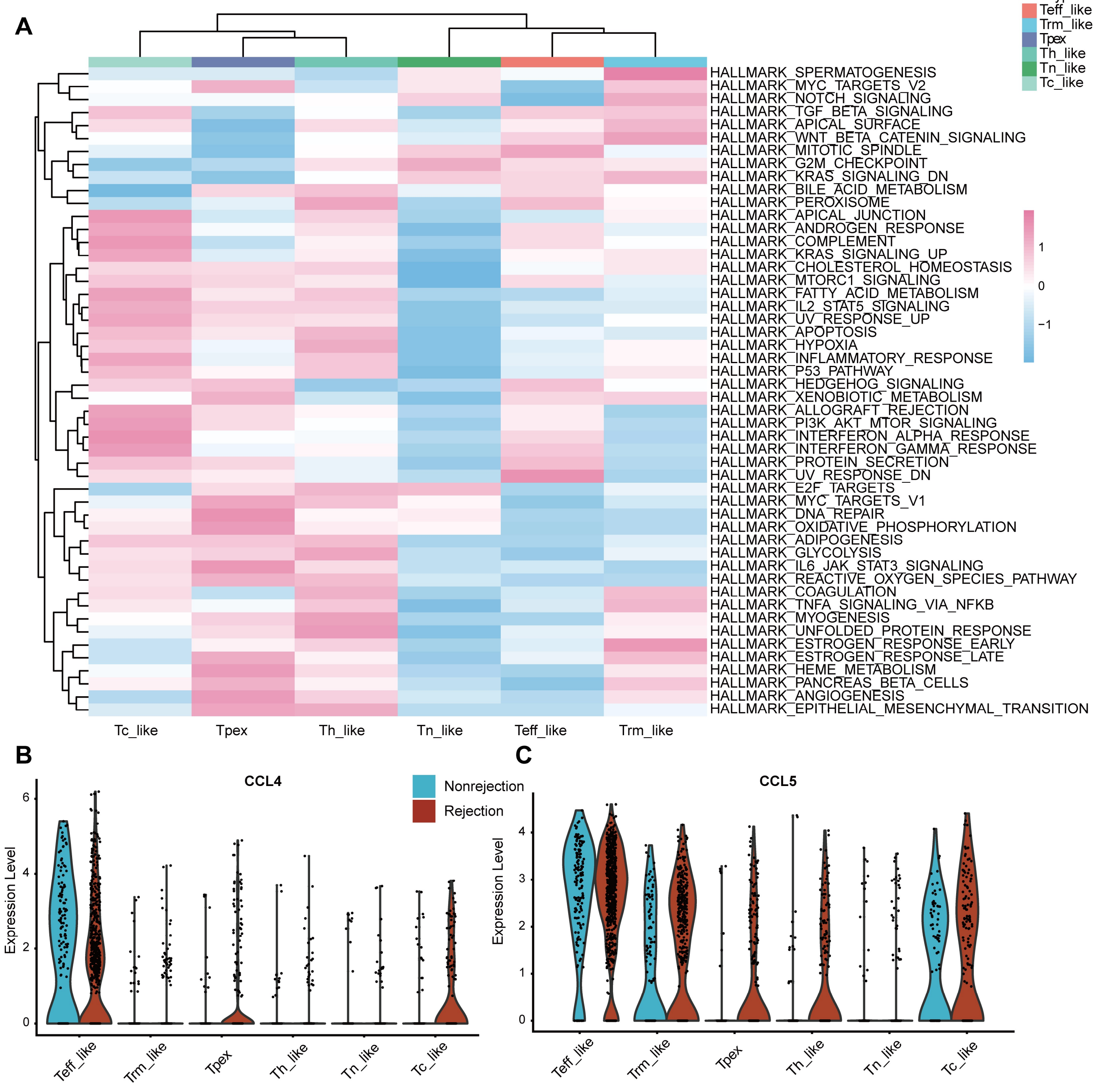

Supplementary Figure 3.

(A) Heatmap showing pathway enrichment differences among taCD4^{+} T subsets.

(B) Expression of CCL4 in taCD4^{+} T subsets.

(C) Expression of CCL5 in taCD4^{+} T subsets.

Supplementary Fig. 4

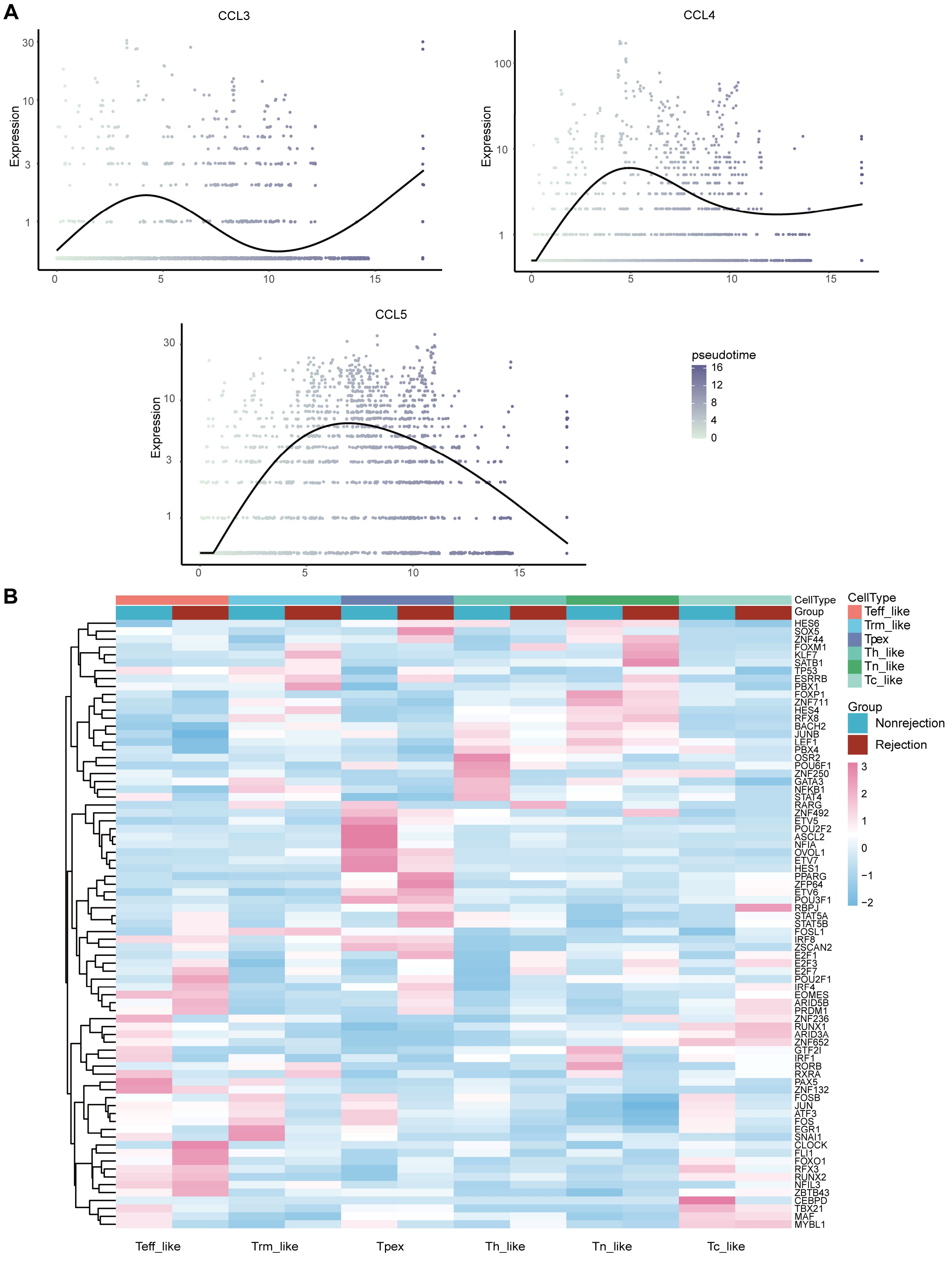

Supplementary Figure 4.

(A) CCL3, CCL4, and CCL5 show peak expression during the early to middle stages of pseudotime.

(B) Heatmap showing transcription factor activity across taCD4^{+} T subsets. Differential activity is observed even within the same cell type across different groups.

Supplementary Fig. 5

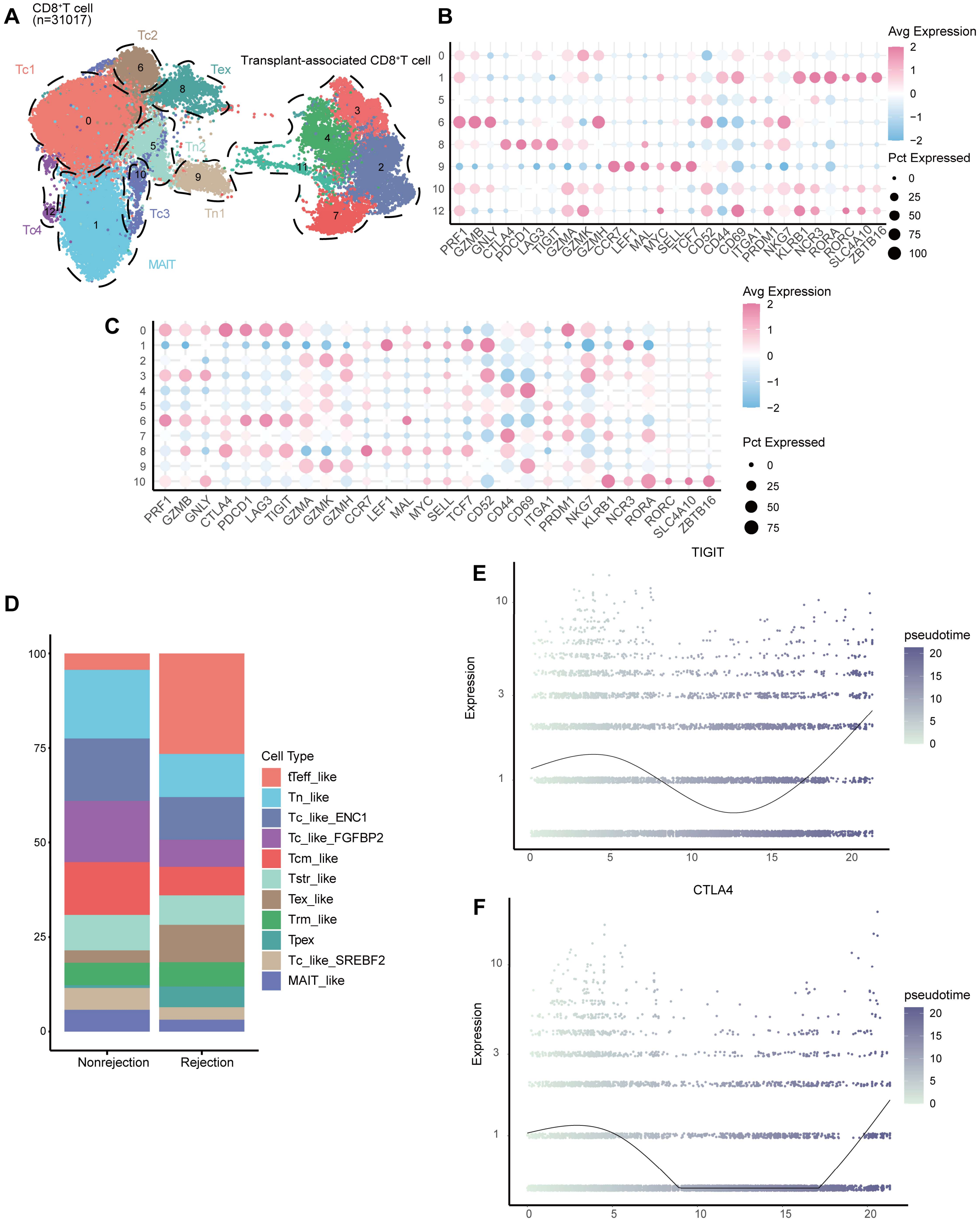

Supplementary Figure 5.

(A) UMAP visualization of CD8+ T cell subsets colored by clusters.

(B) Dot plot showing expression of canonical CD8+ T cell marker genes.

(C) Dot plot showing expression of more canonical taCD8^{+} T cell marker genes.

(D) Bar plot showing the proportions of taCD8^{+} T subsets between the rejection and nonrejection groups.

(E, F) Pseudotime expression dynamics of TIGIT and CTLA4 in taCD8^{+} T subsets.

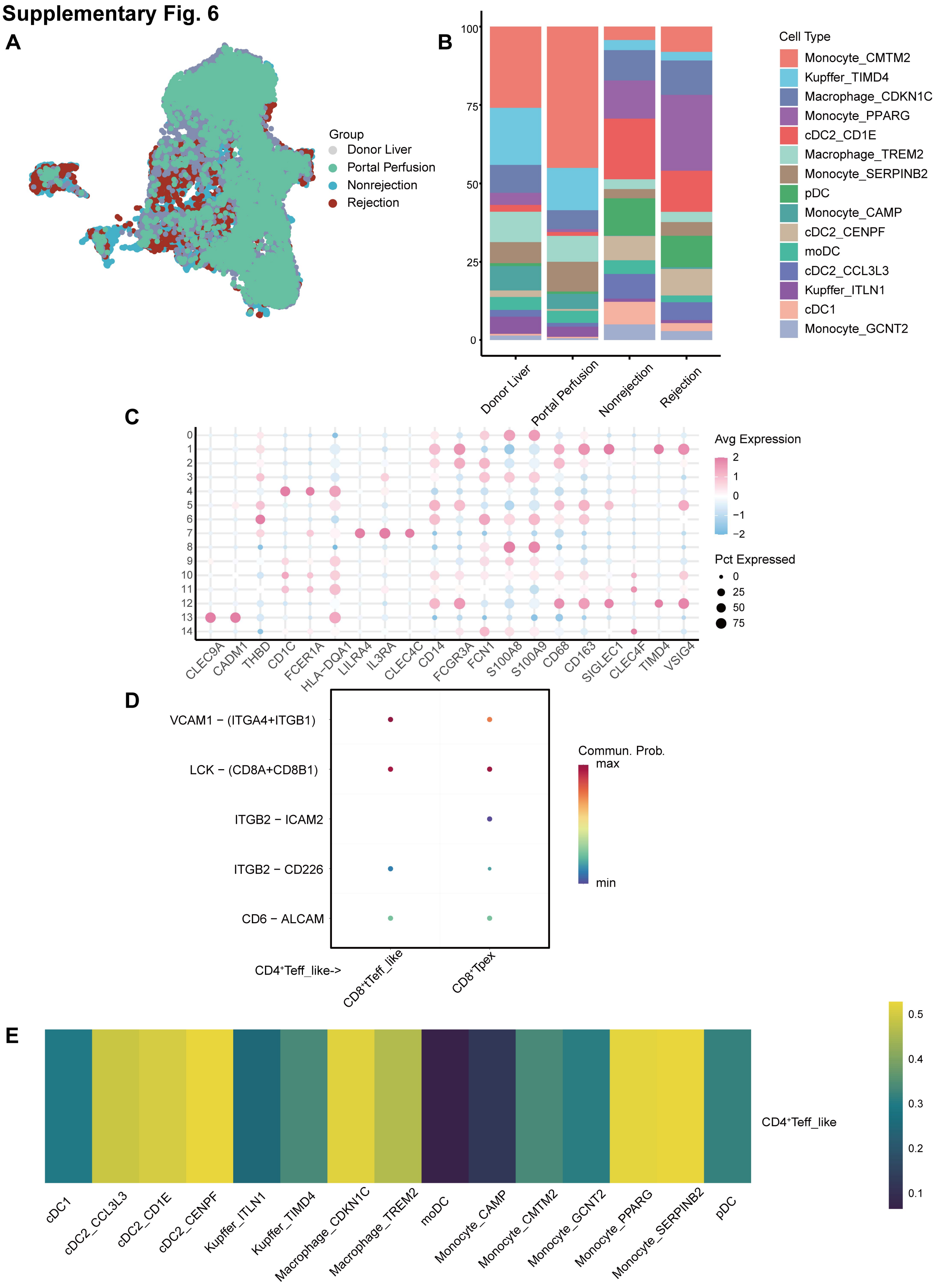

Supplementary Figure 6.

(A) UMAP visualization of myeloid subsets colored by group.

(B) Bar plot showing the proportions of myeloid subsets in each group.

(C) Dot plot showing the expression of canonical marker genes across myeloid subsets.

(D) Ligand-receptor interaction analysis between the $CD4^{+}$Teff_like subset with $CD8^{+}$ Tpex and $CD8^{+}$ tTeff_like subsets.

(E) Communication strength analysis between the $CD4^{+}$Teff_like subset and myeloid subsets.

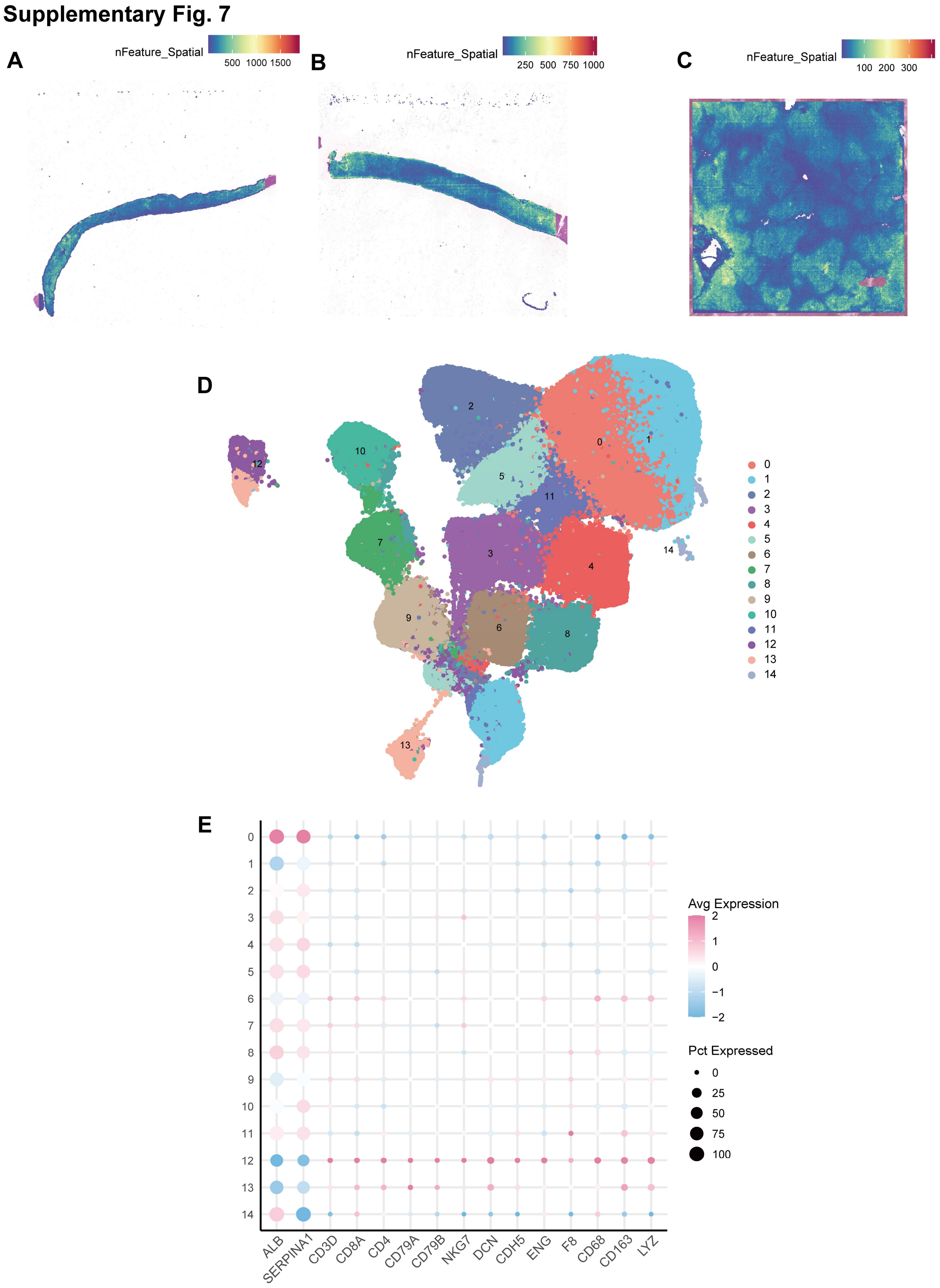

Supplementary Figure 7.

(A-C) Quality control of ST (Visium HD) data for three samples.

(D) UMAP visualization of ST (Visium HD) data colored by clusters.

(E) Dot plot showing expression of canonical marker genes across spatial bins.

Supplementary Fig. 8

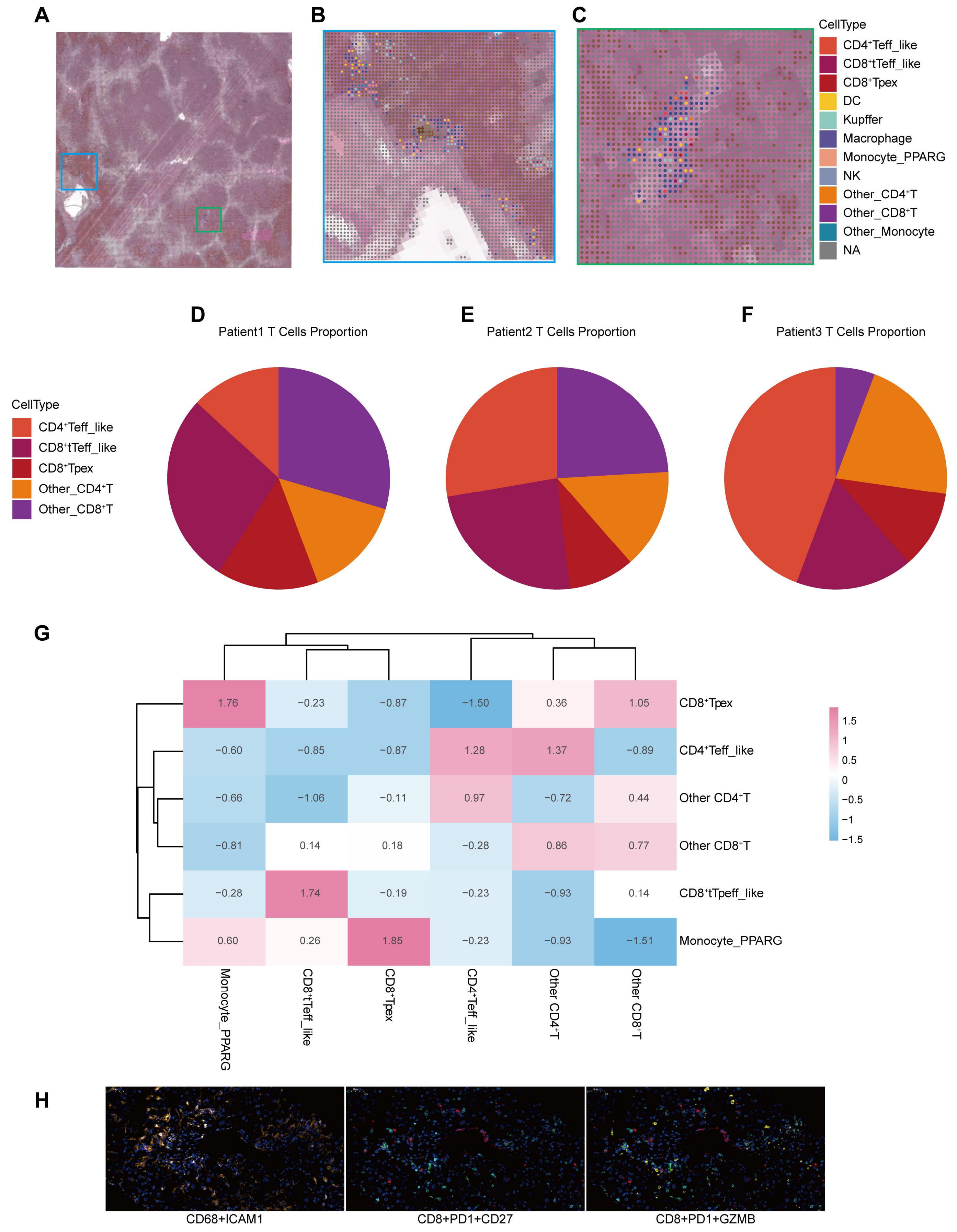

Supplementary Figure 8.

(A-C) Deconvolution results of Patient 3 in ST.

(D) Pie chart showing the proportion of T cell subsets in Patient 1.

(E) Pie chart showing the proportion of T cell subsets in Patient 2.

(F) Pie chart showing the proportion of T cell subsets in Patient 3.

(G) Neighborhood analysis between Monocyte_PPARG and T cell subsets.

(H) MpIF showing the spatial distribution of CD68+ICAM1, CD8+PD1+CD27, and CD8+PD1+GZMB in the portal area of rejection grafts.
